# Impact of COVID-19 Pandemic on Daily Mobility of the Elderly Living in Small Cities in Lodz Province

**DOI:** 10.3390/ijerph20095752

**Published:** 2023-05-08

**Authors:** Marta Borowska-Stefańska, Maxim A. Dulebenets, Michał Kowalski, Filip Turoboś, Szymon Wiśniewski

**Affiliations:** 1 Institute of the Built Environment and Spatial Policy, University of Lodz, 90-142 Lodz, Poland; 2 Department of Civil & Environmental Engineering, Florida A&M University-Florida State University (FAMU-FSU), Tallahassee, FL 32310, USA; 3 Institute of Mathematics, Lodz University of Technology, 93-590 Lodz, Poland; 4 Research Center for European Spatial Policy and Local Development, University of Lodz, 90-142 Lodz, Poland

**Keywords:** daily mobility, elderly, COVID-19, CATI, statistics testing, small cities

## Abstract

The article presents a study into the impact that the COVID-19 pandemic had on the daily mobility of those over 60 residing in small towns in the Lodz Province. The study determines the impact on the trip destination, trip frequency, preferred means of transport, distance and duration of trips, and length of the target activity. To achieve these objectives, a survey was conducted using the CATI technique (Computer Assisted Telephone Interviewing), which comprised 500 residents of small towns in the Lodz Province aged 60+, who were divided into three classes of small towns (by population size). In order to determine the impact of the COVID-19 pandemic on the daily mobility of those over 60, the tools the authors decided to use descriptive statistics and hypothesis testing. Overall, the pandemic was found to have had only a minor impact on the changes in transport behavior of those over 60 in small towns. Only 9% of respondents declared any effect on their daily mobility. The impact mainly involved a reduction in travel time and frequency, primarily among the oldest residents. Since a low level of daily mobility leads to low social activity, especially for the elderly—with a consequent sense of loneliness or even depression-towns should take measures to improve the already poor situation, one that has been further exacerbated by the pandemic.

## 1. Introduction

The COVID-19 pandemic caused a major global economic upheaval. Due to its highly contagious nature, drastic measures were adopted to restrict people’s daily mobility leading to changes in mobility patterns, as people were expected to stay at home, with mobility restricted to essential activities only. The most vulnerable populations, both in terms of susceptibility to coronavirus and the knock-on effects on mental health, were those over 60.

The impact of the COVID-19 pandemic on various aspects of life has been extensively addressed in the literature, including its effects on the economy [1], the environment—air quality in particular [2,3,4], and transport behavior [5]. Considerably less attention, however, has been devoted to the daily mobility of those over 60, as noted by Liu et al. [6]. The elderly were recommended to stay home during the pandemic, yet basic needs must be met. In addition, one should remember that mobility is an essential aspect of the quality of life [7,8,9]. Low levels of daily mobility lead to low social activity among senior citizens and, consequently, to a sense of loneliness or even depression [10]. The shift in the modal split was caused by the pandemic and the resultant reduction in public transport use in favor of walking or the car. This has resulted in the loss of some bus routes, further worsening accessibility to mass transit in small towns and rural areas, thereby leading to transport exclusion for many. Since transport exclusion is an increasingly pressing issue for the elderly, the pandemic’s impact on the everyday mobility of those over 60 and on small towns and rural areas implementing the principles of sustainable urban mobility is an issue worth investigating.

The subject of the impact of the COVID-19 pandemic on various spheres of human life has become the subject of research interest of a wide range of scientists representing various fields and disciplines of science. However, this does not change the fact that the “depth” of individual research threads has not been fully penetrated. While at first glance, the presented study may be part of a broad trend of pandemic research, in the authors’ opinion, it concerns a social group with such unique characteristics that it is worth analyzing. Older people who live in small towns are a group with special needs. Moreover, the pandemic is only one of the possible determinants of limiting spatial mobility. The fact that the threats related to COVID-19 seem to be under control does not mean that it is not worth analyzing changes in spatial mobility in the face of a stressor limiting displacement. Older people who live in small towns are a group with special needs. Moreover, the pandemic is only one of the possible determinants of limiting spatial mobility. The fact that the threats related to COVID-19 seem to be under control does not mean that it is not worth analyzing changes in spatial mobility in the face of a stressor limiting displacement. Knowledge about the features of transport behavior during the pandemic is not only used to shape social policies (including mobility) related to such drastic events. It can also be very useful in circumstances of other origin—e.g., inflationary pressure and the accompanying increase in the cost of living of the population.

This article describes a study into the impact of the COVID-19 pandemic on the daily mobility of those over 60 residing in small towns and determines its impact on the following: trip destinations and frequency, preferred means of transport, distance and duration of trips, and length of the target activity.

## 2. Literature Review

### 2.1. Daily Mobility among Those over 60 in Small Cities and Rural Areas (Transport Exclusion)

The problems that those over 60 residing in small towns and rural areas experience are gaining attention from international organizations, scientists, and governments in many countries. This rapidly growing interest stems, inter alia, from the recognition of how life outside large cities is for those over 60. Improving life for those over 60 in small towns and rural areas is a process that poses a huge global challenge; therefore, it is vital to conduct research on this particular group [11]. The growing number of senior citizens will also have more and more impact on the transport sector [12,13,14,15], which faces particularly severe problems outside large cities. Mobility is a fundamental need of people of all ages, inseparably associated with independence, autonomy, and quality of life [8,16,17,18,19,20,21,22]. There is a large number of studies that investigate the impact of aging on the transport behavior of city dwellers. These have shown that as the population ages, there is a reduction in mobility [23,24,25]. This process is further exacerbated in small towns and rural areas, where traveling outside town or village involves significantly more time, effort, and, oftentimes, also money.

By and large, those over 60 who remain mobile are treated as a homogeneous group, despite the fact that they are, in fact, very heterogeneous in terms of their social and economic status, health, fitness, lifestyle, motivation to travel, and place of residence (e.g., big city, small town, rural area).

The mobility of those over 60 in small towns and rural areas is mainly determined by their health [7]. The issue of th mobility of those over 60 was first debated in the early 1970s. At that time, studies focused on their use of buses [26,27,28,29]. Another mobility determinant is that public transport is often not suitable for those over 60 [16].

Getting those over 60 to use public transport more frequently is beneficial for society as a whole. People, in general, are accustomed to traveling by car, which is often the only mode of transport available outside large cities. However, due to the physical impairments associated with aging (including diseases) that typically occur in old age, those over 60 frequently pose a danger on the roads, and their actions often lead to accidents [19]. For that reason, this group, in particular, should be encouraged to use public transport to maintain the mobility that is an essential element in the quality of life. Research shows that the lack of transport alternatives in rural areas and small towns makes those over 60 reluctant to stop driving, as they identify the car with a sense of autonomy and independence [30,31,32].

It is clear that those over 60 residing in small towns and rural areas are at risk of social exclusion. This can be for many reasons, for instance: transport-related, lower physical capability, health issues, and financial and digital exclusion. Given that the picture emerging from numerous studies presents walking as a lesser form of spatial mobility for the elderly, it seems imperative to focus on those elements in small towns and rural areas that impact their mobility, including access to toilets, regular benches, and appropriate street lighting [33]. These would increase the popularity of walking in the overall travel volume.

As regards increasing the use of public transport, making public transport more user-friendly for this section of society should be considered (e.g., launching dedicated minibusses that drop those over 60 close to where they reside). Such measures could affect future transport preferences and, above all, the decision to not use the car. The literature also advocates that obstacles posing a danger to those over 60 should be removed from public spaces.

Studies by Gell et al. [34] indicate that it is essential for those over 60 to be able to walk to amenities and recreational facilities. Thus, in order to promote the spatial mobility of those over 60 in small towns, the services that they need should be within walking distance of the elderly’s place of residence. Walking is also beneficial for those over 60, both as regards fitness and costs [35].

### 2.2. Changes to Daily Mobility during the COVID-19 Pandemic

Once COVID-19 had been recognized as a global public health concern, many countries struggled to prevent the appearance and subsequent local transmission of SARS-CoV-2, i.e., the virus that causes COVID-19 [36]. This resulted in most countries in the world introducing a range of restrictions, affecting not only business activity but also transport, traveling, and people’s daily mobility [37,38,39,40]. A variety of severe restrictions (including policies on staying at home; schooling, public institutions and workplaces going remote; cancellation of mass events and public gatherings, as well as restrictions on public transport) affected around 90% of the global population, contributing to an overall reduction in mobility on an unprecedented scale [41,42,43]. The greatest relative drops in mobility were mainly seen in large cities, with a slightly different situation in small towns and rural areas. The pandemic and the accompanying restrictions did not paralyze mobility in these areas so severely due to the often lower awareness of the residents, the already low baseline level of mobility, and the lower enforcement of the introduced restrictions over the population there.

As demonstrated by Vannoni et al. [44], the severity of the government’s response to COVID-19 strongly decreased mobility. The implementation of non-pharmaceutical countermeasures intended to reduce the spread of severe acute respiratory syndrome coronavirus affected people’s transport behavior, especially daily mobility [45,46,47,48]. In Europe, passenger traffic fell by as much as 90% [42], a decline that was most acute for public transport, as demand for commuting plummeted and transport operators reduced their services [49]. Viewed from this perspective, residents of small towns and rural areas were relatively unaffected by the impact that the pandemic had on the use of public transport since the transport modal split is based largely on the car there (including enforced car dependency). In addition, the perception of public transport with regard to its safety deteriorated, as confined and crowded places, including the transport hubs previously frequented by throngs of commuters on a daily basis, were considered potential sites for the spread of coronavirus [50,51].

There are two major approaches to studying the issue of mass transit in relation to the COVID-19 pandemic. On the one hand, researchers analyze the impact of the transport sector on the spread of COVID-19, with the majority of studies looking at the way in which changes in spatial mobility affected the spread of the COVID-19 pandemic [52]. Zheng et al. [53] indicate that COVID-19 cases connected to transport modes and hubs are a key factor in the spread of the pandemic, Linka et al. [54] imply that unrestricted mobility would have significantly accelerated its spread, particularly in central Europe, Spain, and France. According to Musselwhite et al. [55], public transport plays a major role in the spread of infectious diseases, and thus, attempts to curb the spread of infection by reducing public transport use can curb the rapidity of transmission. On the other hand, other researchers examine how the COVID-19 pandemic and the accompanying national and local restrictions reduced the demand for mass transit and adversely affected the state of transport in general [56]. Aloi et al. [57] studied the impact of pandemic-related restrictions on the mobility of urban populations, establishing that the overall mobility in Santander (northern Spain) decreased by 76% and the number of public transport users dropped by as much as 93%.

The existing pool of knowledge on spatial mobility during the COVID-19 pandemic indicates that its impact on public transport led to changes in people’s transport behavior, with a resulting impact on sustainable mobility [58,59]. As evidenced by Przybylowski et al. [46], the pandemic triggered dramatic changes in mobility, causing, amongst others, a drop in public transport and an increased reliance on the car. Pandemic-related changes in the modal split were also recognized by De Haas et al. [60], Beck and Hensher [61], and Jenelius and Cebecauer [49], who established that the pandemic resulted in a rise in the popularity of both car and non-motorized modes of transport (cycling, walking) at the expense of public transport, which was particularly evident in data on daily mobility. Thus, the car, which was already the most popular choice in urban areas and small towns in the pre-pandemic period, became even more dominant.

A growing number of publications have shown a keen interest in the impact of the coronavirus pandemic on mobility, which makes it possible to compare the impact of pandemic-related restrictions on mobility on an international scale [44,46,62,63]. In addition, this issue is worth considering when conducting comparative analyses of transport in large cities, small towns, and rural areas. In this respect, it is vital to monitor the temporal changes in travel behavior induced by the pandemic [64,65]. Another extremely salient aspect is transport policy, one aspect of which is to eliminate inefficiency in managing transport demand and supply for all stakeholders [66]. A major research theme emerging in the international literature is the assessment of the impact the pandemic had on population distribution, resulting from the growing importance of remote working, e-services, e-government, etc. [67,68,69]. From this perspective, another subject worth pursuing is digital exclusion, which especially affects the elderly in rural areas and small towns.

## 3. Methods

### 3.1. Study Area

**Figure 1:**
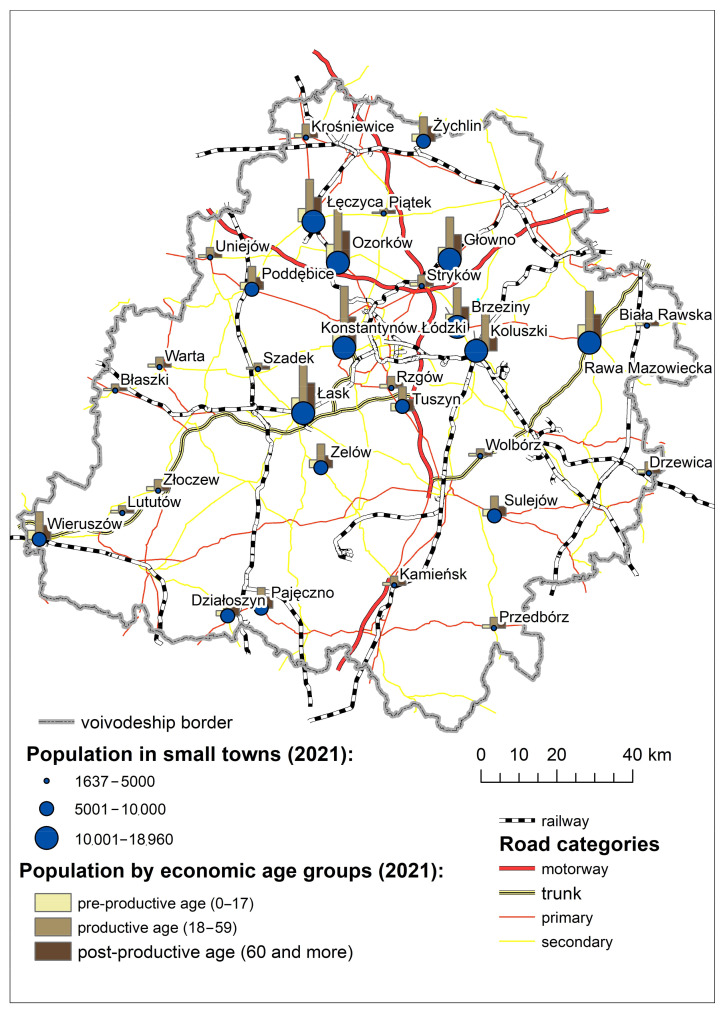
Population of small towns in the Lodz Province by economic age group.

The ‘small town’ is a concept generally formulated on the basis of the legal status of a settlement unit and basic demographic statistics (number of inhabitants). In Poland, a town is any settlement unit with a predominantly compact built-up area and a predominantly non-agricultural function that has been granted town rights. Small towns are generally considered those with a population of 20,000 or below. The criterion applied here is not universal but specific to the Central European settlement structure. Small towns are inhabited by 12.85% of Poland’s population, and a small-town nature lies not in numbers but in the specificity of these places and the social relationships there [70]. In 2021, there were 31 small towns in the Lodz Province (Figure 1). Since these small towns are quite diverse in terms of their population, it was advisable to develop a preliminary classification for these settlement units. Three subsets can be distinguished (up to 5000, between 5000 and 10,000, and from 10,000 to 20,000 inhabitants) where the internal structure of demographics shows little variation (Table 1). These subsets correspond to the categories commonly adopted in administration and statistics [71].

By and large, the bigger a small town, the older the population. One effect of this is the higher feminization rate among those over 60 and the negative natural population change observed in the largest small towns. The only small towns where a natural population change was observed were two small settlements that had regained town rights relatively recently: Rzgów (in 2006) and Wolbórz (in 2011).

**Figure 2:**
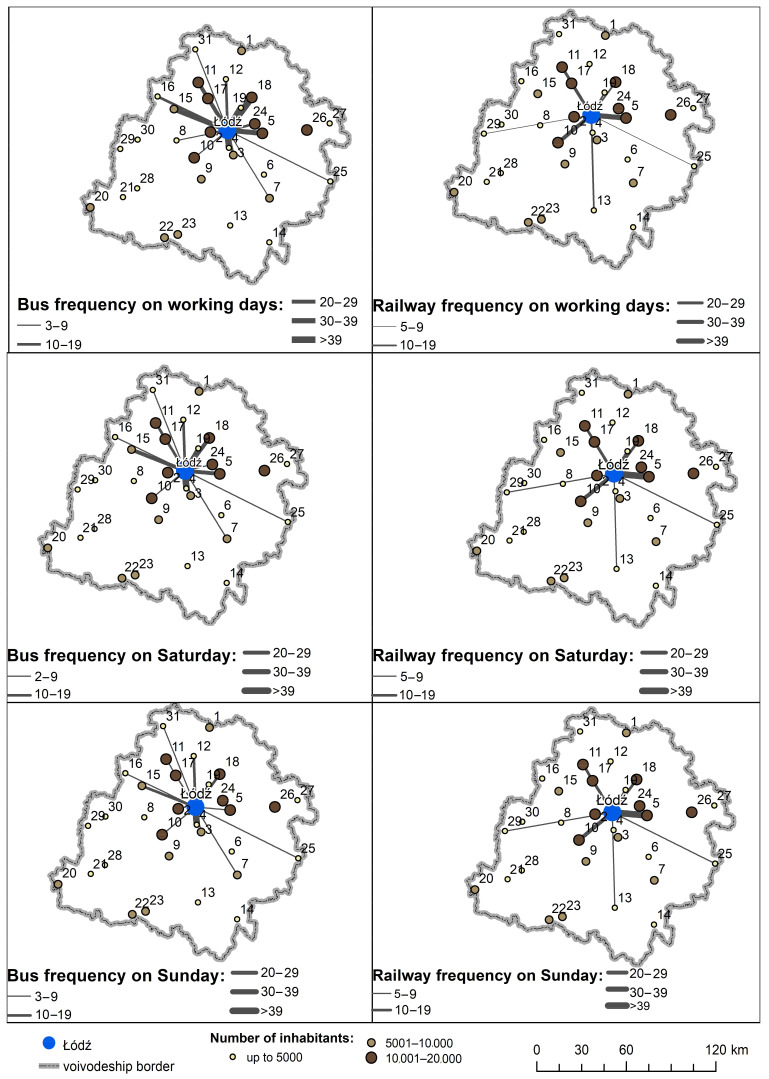
Frequency of public transport service from small towns in the Lodz Province to the city of Lodz. 1—Żychlin; 2—Konstantynów Łódzki; 3—Tuszyn; 4—Rzgów; 5—Koluszki; 6—Wolbórz; 7—Sulejów; 8—Szadek; 9—Zelów; 10—Łask; 11—Łęczyca; 12—Piątek; 13—Kamieńsk; 14—Przedbórz; 15—Poddębice; 16—Uniejów; 17—Ozorków; 18—Głowno; 19—Stryków; 20—Wieruszów; 21—Lututów; 22—Działoszyn; 23—Pajęczno; 24—Brzeziny; 25—Drzewica; 26—Rawa Mazowiecka; 27—Biała Rawska; 28—Złoczew; 29—Błaszki; 30—Warta; 31—Krośniewice.

**Figure 3:**
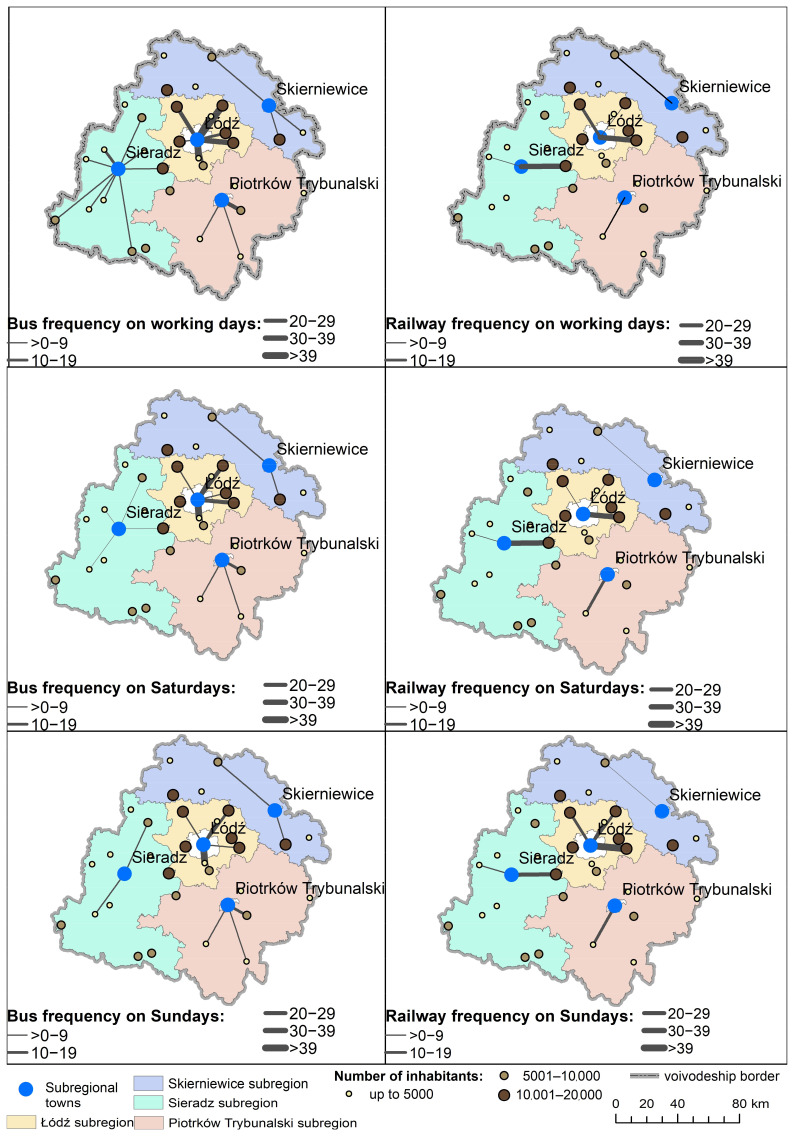
Frequency of public transport service from small towns in the Lodz Province to sub-regional centers.

Only a limited number of small towns have sufficient direct public transport connections to the regional capital (Figure 2). Namely, only those in the central part of the region have a sufficient number of direct connections. The remaining towns find themselves in a transport desert since, even on weekdays, they are not linked by public transport at a usable frequency, even to the sub-regional centers (Figure 3).

### 3.2. Questionnaire Survey

**Figure 4:**
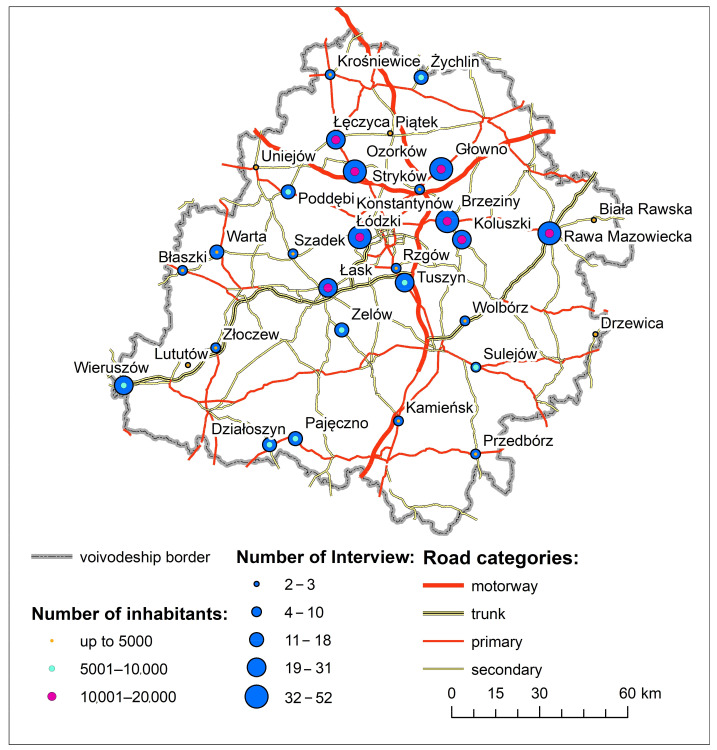
Number of interviews conducted in small towns in the Lodz Province.

In order to assess the impact of the COVID-19 pandemic on the daily mobility of the 60s and more residing in small towns in the Lodz Province, a survey was conducted using the CATI (Computer Assisted Telephone Interviewing) technique involving 500 inhabitants of small towns in the region. The inclusion criteria for this sample collection consisted of age (60 and more) as well as the housing town (small towns of the Lodzkie province). The only exclusion criterion applied was the refusal to conduct CATI. For the purposes of this study, the three classes of small towns were as follows: up to 5000 (15 towns), from 5000 to 10,000 (8 towns), and between 10,000 and 20,000 (8 towns) inhabitants. The applied classification aggregates settlements that have the most similar profile to make the three sets as homogeneous as possible. The criterion of population size has been applied, inter alia, by Kiełczewska-Zaleska [72,73,74,75,76]. The number of interviews conducted varied depending on the class of small town (i.e., in towns with up to 5000 inhabitants, a total of 91 interviews were conducted; in towns with 5001 to 10,000 inhabitants, 125 interviews; and in towns with 10,000 to 20,000 inhabitants, 284 interviews) (Figure 4). A total of 0.8% of those 60 and over in these settlements were surveyed. The research tool applied was an interview questionnaire consisting of two main parts: the respondent’s particulars and questions on the impact that the COVID-19 pandemic had on their daily mobility, including trip frequency, mode of transport, trip distance and duration, and the duration of the target activity. The survey was conducted in October 2022, while the questions concerned daily mobility in the previous month (September 2022).

Telephone testing has its limitations, which have already been extensively discussed in the literature. One of the fundamental disadvantages, presented theoretically, is the digital exclusion and diversification of access to the telephone. In the case of small towns (and even rural areas) in Poland, access to the telephone is not a barrier. What is more, the interviewers were properly prepared to work with the elderly; they were sensitive to the specific characteristics of these people. At the time of the study, the pandemic situation in Poland was not yet clear. Face-to-face testing would create a lot of uncertainty and resistance to meetings with interviewers. Therefore, a telephone survey is the best possible solution; it allows you to avoid the risks associated with direct contact and, at the same time, gives the respondent the opportunity to participate in the survey actively. The latter element is especially important in the case of the elderly.

### 3.3. Variable Dependency Analysis

In order to determine the impact of the COVID-19 pandemic on the daily mobility of the elderly, the tools we have mainly resorted to were descriptive statistics and hypothesis testing. Because the second part of the questionnaire consisted mainly of branching questions, we have decided to split the dataset into two subgroups—those elderly whose daily mobility has been affected by the COVID-19 pandemic and the remaining ones. In the latter subgroup, we have restricted ourselves to descriptive statistics to characterize their profile and raise hypotheses on the reasons why their traveling habits have not changed because of the pandemic. We also used correlation tests (Spearman ρ/Kendall τ correlation tests) to determine whether correlations among the demographic variables in this group are statistically significant. 

As far as the second group is concerned, we utilized descriptive statistics to outline the exact effects of the pandemic on their mobility. The results were, at least in some places, somewhat surprising. Then, via the use of statistical tests, we tried to elucidate which demographic characteristics of the respondents were related to the changes they pointed out in the questionnaire—which, again, were mainly correlation tests. 

Lastly, we have tried to compare several characteristics of the respondent groups and see whether we encounter any significant differences. Due to the fact that the data violated the normality assumptions, we could not use typical tests such as the *t*-test or Welsh test. Therefore, we had to resort to Mann–Whitney–Wilcoxon test in such cases. 

#### Data Preparation

Due to the nature of the questionnaire answers, we had to preprocess the data to some extent. We have decided to impute the missing values of the age variable by the center of the age interval selected in the subsequent question. A similar technique was used to replace the income intervals by their respective centers. We have also used a numeric scale to replace the descriptions of educational backgrounds. As far as the current occupation is concerned, we have used one-hot encoding. We have also created summary variables for changes to the elderly people’s mobility induced by the pandemic. Because we had to take into account people who experienced both increases and decreases in certain travel characteristics, we have used indicative variables in such cases (where pair of zeros denoted no changes in a given category, pair (1,0) described decreases in certain travel scenarios, (0,1) denoted the increases in such characteristic for certain mobilities and (1,1) was reserved for the respondents who have encountered both rises and falls in said category). 

### 3.4. Limitations and the Implication of the Study

The study was planned and conducted in a way that minimized imperfections. However, the limitations of the study could not be completely eliminated. Telephone contact with respondents may be a kind of limitation. However, this issue has already been discussed previously. The inclusion in the analysis of settlement units from only one region, whose community of elderly settlements may be specific, can also be considered a limiting factor. However, the Łódź Voivodeship is a region whose settlement network has been shaped by numerous influences of historically diverse origin. This is because it is a “border” area in relation to the neighboring, shaped historical and geographical lands. This diversity within the Łódź Voivodeship significantly reduces the effect of homogeneity of research units. Small towns in the Łódź Voivodeship are a heterogeneous collection in many respects. Therefore, the obtained results can be regarded as highly universal. This feature also allows us to talk about the high level of applicability of the obtained results. They can be helpful in shaping transport and social policies (including activating older people and sustainable mobility in areas outside large urban centers). They can be shaped both in the face of “normal” circumstances as well as in the event of the reappearance of factors limiting the spatial mobility of the population (including the elderly) of external origin, i.e., not resulting from free decisions of people. During the “normal” time, they will help to meet the special needs of the elderly, maintaining a sense of independence that is very important for this group. This issue is very difficult to include in the mobility policy outside large cities because, in areas of lower population concentration, there is naturally less competition from public transport. Elderly people are almost forced to use only private car transport (forced motorization). With age, their ability to drive is getting worse, and this has a destructive effect on their sense of agency and makes them excluded on many social levels. In periods of a stressor limiting mobility, thanks to the conducted research, it becomes possible to introduce intervention solutions that will not allow for a drastic degeneration of the elderly’s mobility and thus prevent the acceleration of their exclusion from active participation in the life of the local community.

## 4. Results

### 4.1. Description of Respondent Characteristics

The study involved 500 respondents residing in small towns in the Lodz Province, whose characteristics are presented in Table 2. Demographic features such as the gender and age of the respondents are similar to those of the overall population of those over 60 in small towns.

### 4.2. Trip Destination and Frequency

**Figure 5:**
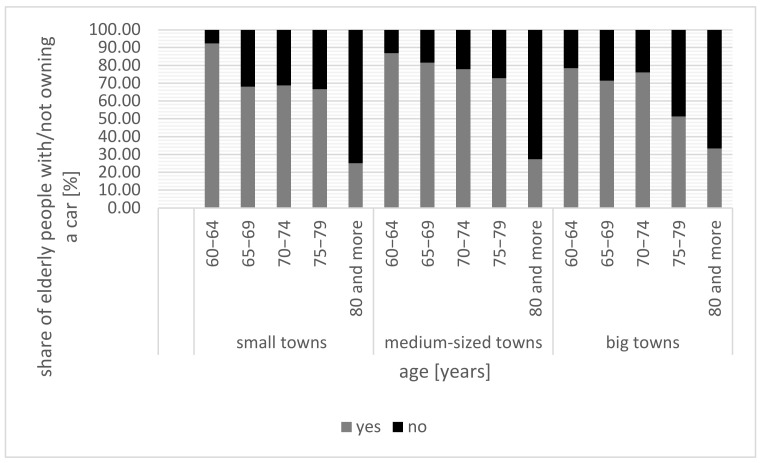
Car ownership among those over 60 by age and small town category.

When analyzing daily mobility, it is important to determine whether a given household has access to a car. There is a significant (primarily age-dependent) diversity in this respect among the respondents. Car ownership is highest among respondents aged 60–64; on average, 83.33% of the respondents within this age bracket have a car in their household across all small-town categories. However, it should be emphasized that this percentage (for ages 60–64) is highest in the smallest towns (up to 4999 inhabitants)—92.31%, and lowest in larger towns (from 10,000 to 20,000 inhabitants)—78.38% (Figure 5).

Analyses of the destinations for the respondents reveal that the most popular motivation behind their daily mobility is shopping (86.8%). Since a significant percentage of the respondents are neither professionally active nor in education, they did not list these destinations among their main trip motivators. As many as 80.4% no longer work (predominantly living on disability benefits or a pension), and in addition to obligatory motivations, it is shopping that remains the most common purpose behind traveling [77].

**Figure 6:**
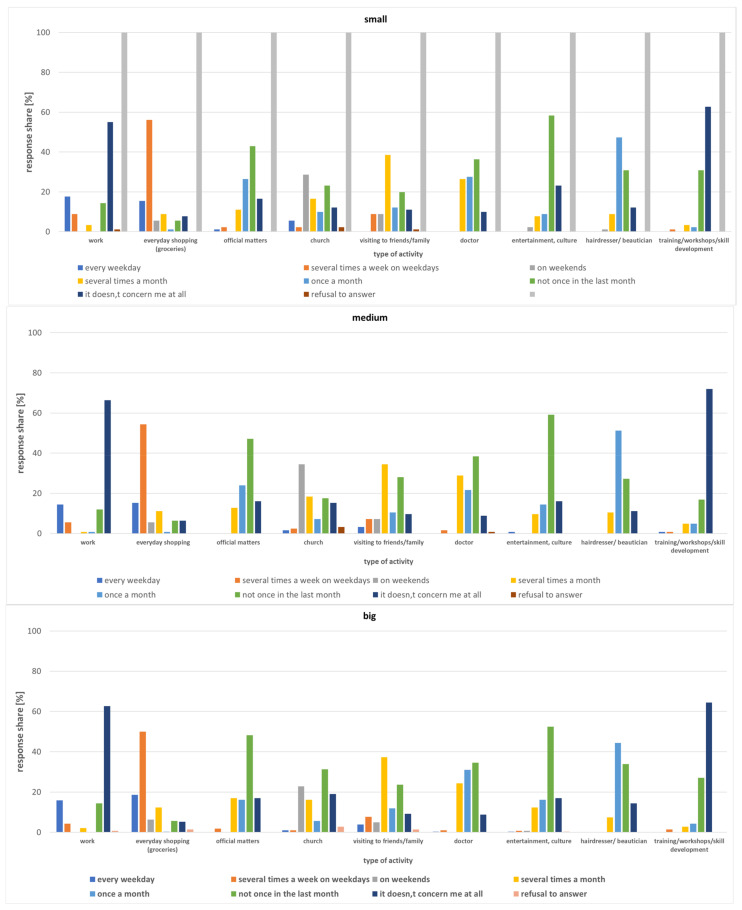
Trip frequency among those over 60 by destination and small town category.

**Figure 7:**
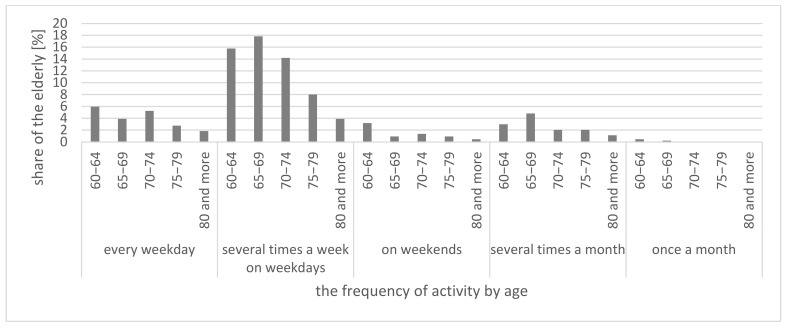
Trip frequency among those over 60 residing in small towns in Lodz Province for grocery shopping by age.

Those over 60 in small towns within the Lodz Province go shopping several times a week (on weekdays) (59.73% of respondents) (Figure 6 and Figure 7). As for visiting friends or family, most respondents selected the answer: several times a month (56.27%). Approximately 50% of those who go to church indicated that they do so at weekends. More than half of respondents also traveled to seek medical care in the said month (September 2022), with the majority stating it was a single trip (50.91%). For professionally active respondents, commuting to work every day was the most frequent answer (67.52%). Other activities are usually handled once a month (administrative matters, hairdresser, beautician, entertainment, culture, courses, etc.) (Figure 6).

### 4.3. Modal Split

**Figure 8:**
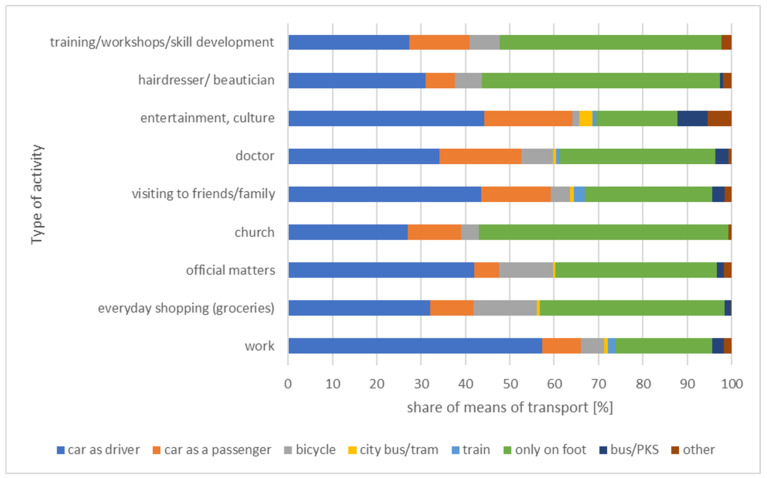
Modal split of individual trips taken by those over 60 in small towns.

The study presented herein shows that those over 60 in small towns most often decide to walk to the church, hairdresser, beautician, etc. The percentage of trips taken solely on foot amounts to 50% or more, while the car plays a more significant role in motivations related primarily to work, leisure, or culture (Figure 8). As regards daily mobility by public transport, for those over 60 in small towns within the Lodz Province, it is of marginal importance.

**Figure 9:**
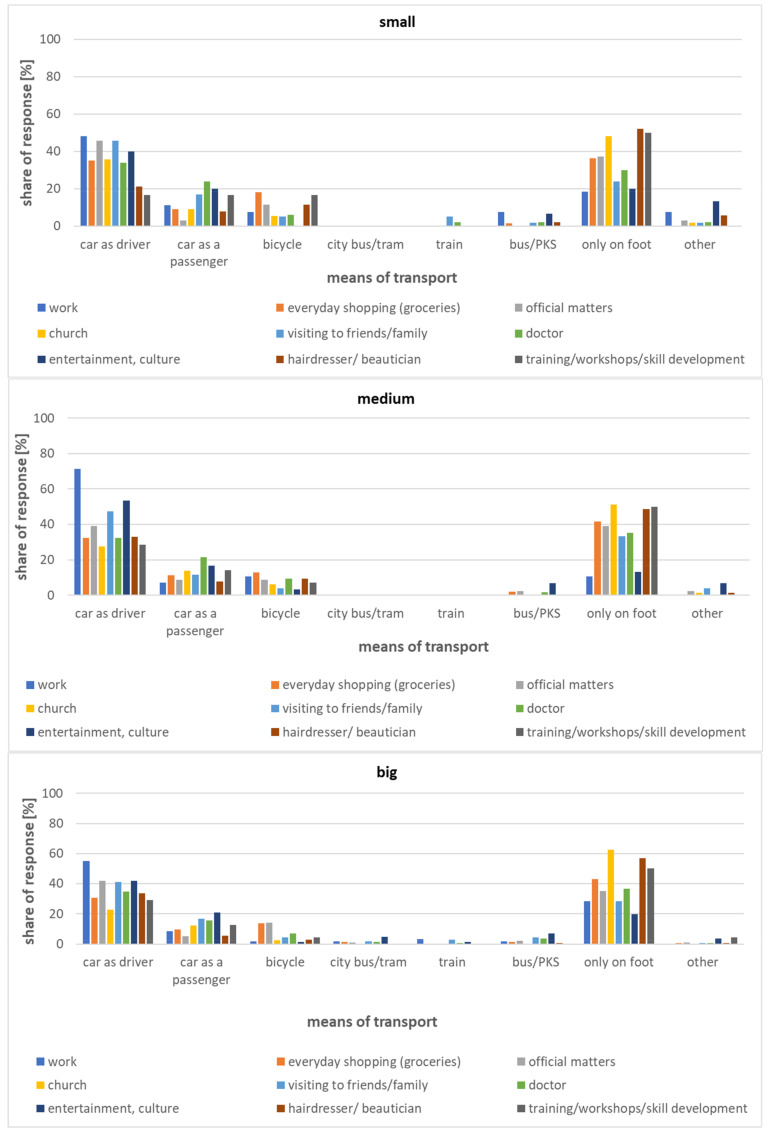
Modal split of individual trips taken by those over 60 for small town category.

As illustrated by the data in Figure 9, the modal split for each class of small towns is similar.

### 4.4. Travel Distance

**Figure 10:**
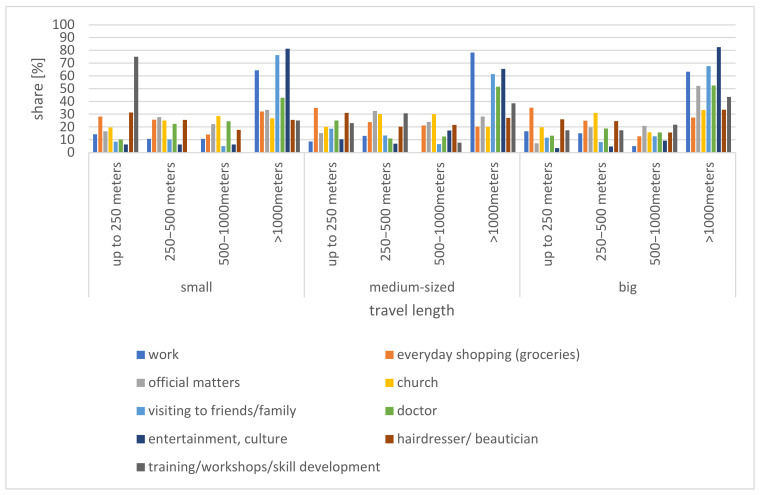
Distances covered during trips taken by those over 60 by small town category.

The results of this study show that those over 60 in small towns within the Lodz Province cover the longest distances for work-related purposes (66.67% of those who commute travel > 1 km), for entertainment and culture (78.63% travel > 1 km), and to visit friends or family (67.72% travel > 1 km). In contrast, covering shorter distances is mainly to visit a hairdresser or beautician (52.03% of these trips are within a distance of 500 m), church (49.25% of trips up to 500 m), and shopping for staples (58.56% of trips up to 500 m), which is true for each class of small town (Figure 10).

### 4.5. Duration of the Target Activity

**Figure 11:**
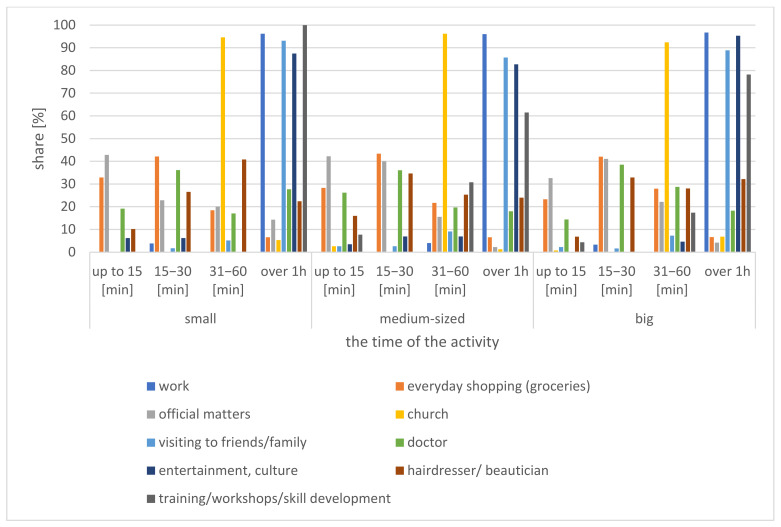
Duration of activities undertaken by those over 60 by small town category.

The study presented herein allows the authors to conclude that those over 60 (who work) devote most of their time to work (96.43% of the professionally active respondents spend more than 1 h on this purpose), to visit friends or family (88.89% of the respondents allocate more than 1 h for this purpose), and for entertainment and culture (91.6% of the respondents dedicate more than 1 h to this) (Figure 11). The least amount of time is devoted to administrative matters, as 74.29% of respondents devote up to 30 min to this purpose, and shopping for staples, with approximately 68.72% allocating up to 30 min for this (Figure 11).

### 4.6. Impact of COVID-19 on the Daily Mobility of Those over 60

Only 9% of respondents declared that the COVID-19 pandemic permanently changed their transport behavior with regard to daily mobility, the majority of those being pensioners (78%). Only 9% of respondents who confirm the impact of the pandemic on their daily mobility remain professionally active, while 11% are not in employment. These people are most likely to have a car in their household (about 71%), with ¼ of them owning more than one vehicle. The pandemic mainly affected the transport behavior of the lower end of our study group (median age: 69 years), among whom men predominated (73%). Those whose daily mobility was affected by the pandemic are statistically better educated than those who did not experience a strong impact from COVID-19 (the *p*-value of the Wilcoxon–Mann–Whitney test amounted to 0.041). The pandemic-induced changes to everyday transport decisions that have become permanently ingrained in society generate both opportunities and problems in planning sustainable transport policies [78]. However, it is difficult to clearly assess the direction of these changes and whether post-pandemic mobility is more sustainable for residents of small towns. A separate issue to consider is how beneficial it is to reduce the mobility of those over 60.

Among those reporting some impact from the pandemic on their daily mobility, approximately 45% indicated that the duration of certain activities dropped; 13% declared the opposite, while fewer than 7% indicated a rise in some activities combined with a drop in others. In general, those residing in multi-family housing were more likely to report a decrease in the duration of an activity (*p*-value equal to 0.0124).

By contrast, the analysis of trip duration itself shows that it remained mostly unaffected by the pandemic. A mere 7% of respondents stated that trip duration had increased for some motivations, and approximately 18% reported the opposite trend. Spearman’s correlation test indicated a relationship between age and a drop in trip duration, i.e., the older the person, the more often they experienced a decrease in trip duration (*p*-value 0.016). The majority of those over 60 who hold a driving license noticed no drops in trip duration, yet this is not a statistically significant observation (*p*-value 0.117). As regards the distance of daily trips, a significant majority of respondents (over 82%) noted a reduction. Those residing in multi-family housing are more likely to report a decrease in the distance of daily trips due to the pandemic, and this observation is statistically significant (*p*-value 0.046).

Finally, 22% of those over 60 respondents stated that the frequency of their daily trips had decreased, while 78% reported no change. Spearman’s correlation test showed a relationship between age and a decrease in trip frequency, i.e., the older the person, the more frequently they experienced a decrease in trip frequency following the pandemic (*p*-value 0.007).

## 5. Discussion

The most important traits that affect the daily mobility among the surveyed groups were gender, age, type of housing, driving license or its lack, number of cars in the household, and income. The results of these analyzes indicate a relatively large percentage of retiree households owning a car. This observation is important in the context that the degree of mobility measured by trip frequency is impacted by a number of factors, one of the most commonly shown in studies being wealth (or income) and the correlated car ownership rate. A study conducted in Sao Paulo by de Vasconcellos [79] indicates that mobility is impacted by both gender, household income, and the resultant car ownership rate. In addition, Zimmerman [80] points out that trip frequency is affected by the family model and the age of the main breadwinner. These studies show that the said three factors are somewhat interdependent.

**Figure 12:**
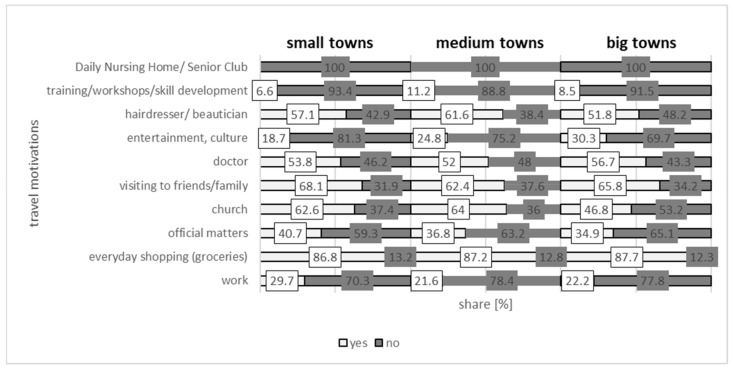
The percentage of those over 60 undertaking (yes) or no (no) daily mobility by group of towns and trip motivation.

The research confirms the observations regarding the frequency associated with those dimensions of daily mobility that are related to shopping. In the UK, around 20% of all trips beginning at home are shopping-related [81] whereas, in Poland, 10.2% of trips are taken for the same reason (9.6% of all trips on weekdays, and 14.2% at weekends), with the percentage in the Lodz Province amounting to 9.5% (9% on weekdays, 12.7% at weekends) [82]. Frequent travel motivations among those over 60 include visiting friends or family (68.1%). None of the respondents indicated an adult day care center or a social club as their destination, while a small percentage (6.6%) indicated courses, workshops, self-development, and also entertainment or culture (18.7%) (Figure 12. These figures are consistent with the results of the study by Borowska-Stefańska and Wisniewski [22] conducted among those over 60 in Lodz. As shown by Czapiński and Panek [83], the most frequent reason for forgoing high-end services is the lack of adequate funds. Our results are also consistent with the results of a study conducted among Poles aged 50+ [84].

Patterns related to modal split may result from several key aspects of the local transport systems. Firstly, small towns are compact settlement systems (often of a concentric nature, as is the case in the study area). As a result, distances between trip destinations and origins are small enough to favor the most time-efficient travel mode in this case, i.e., walking. It is also evident from the curves of ‘there and back’ travel times [85] that walking is most efficient for distances of up to a kilometer. Public transport plays a marginal role as it is ineffective in such places.

The reasons behind the particular structure of the modal split for those over 60 in small towns within the Lodz Province (especially as regards the time-efficiency curve of traveling in relation to the distance to be covered) are confirmed by the analysis of the distances to destinations for different motivations.

Research shows that the impact of the pandemic on mobility depends on the level of education. This may be due to the fact that better-educated people are employed in industries where remote work is possible. Less educated people may engage in activities that require direct participation—for example, in manufacturing processes or in agriculture or construction.

## 6. Conclusions

Although the study established that the transport behavior of those over 60 in small towns within the Lodz Province changed after the pandemic, fewer than 1 in 10 respondents declared its impact on daily mobility in the post-pandemic period (mainly a reduction in travel time and frequency, primarily among the oldest residents). Since low levels of daily mobility translate into limited social activity among this group and subsequent feelings of loneliness or even depression, small towns should take measures to improve the situation of those over 60, which has been further exacerbated by the pandemic.

Within the surveyed population, as many as 80.4% do not work (predominantly those living off a pension), and their daily mobility mainly concerns shopping (several times per week). As regards the time spent, those over 60 spend most of their time visiting friends or family, while those professionally active also commute to work. Shopping, on the other hand, mainly takes less than 30 min. Importantly, when it comes to the daily mobility of those over 60 residing in small towns within the Lodz Province, those destinations that are a short distance away are commonly reached on foot, while longer distances are mainly covered by car.

The results of the conducted study refer to the issue of care for the community and health of the neediest areas (small towns and rural areas). For this reason, analyses of this kind should be considered important, despite the fact that the subject of pandemic research seems to be highly exploited. Despite the growing awareness of the increasing participation of older people in society and the resulting civilizational challenges, rural areas or small towns remain a bit behind the mainstream. The shortage of funds for the implementation of an appropriate policy activating the mobility of the elderly in the peripheral areas of large urban centers is also problematic. The limited offer of higher-order services in rural areas and small towns is also significant. Given the appropriate level of mental and physical health of the elderly, it is necessary to study their mobility also in such special circumstances as the COVID-19 pandemic.

Those over 60 residing in small towns and rural areas are at risk of social exclusion due to, amongst others, their lower physical capabilities, health issues, and financial and digital exclusion. Given that the picture emerging from this study shows that walking could play a major role in the spatial mobility of those over 60, it seems imperative to focus on these elements of the transport infrastructure in small towns and rural areas that impact the mobility of this group [33] in order to increase the popularity of walking in the overall travel volume. However, it is public transport—whose role in small towns still remains only marginal—that requires the most substantial changes in these areas. This marginality was further exacerbated by the pandemic when there was a very high potential risk of virus transmission in mass transit [55,86]. As a result, it was those over 60 in particular (a group at increased risk of morbidity and mortality following coronavirus infection) who were advised against using public transport. The pandemic affected public transport services detrimentally: many unprofitable connections were discontinued (mainly in rural areas and small towns), whereby some people either abandoned certain motivations—thus condemning themselves to transport exclusion—or began to use the car more frequently [87]. To achieve a more balanced transport spectrum, measures must be taken in the realm of transport policy that will impact transport preferences in the future, especially to curb the use of cars in small towns. It would also be valuable to study the impact of COVID-19 pandemic on the quality of life of the elderly.

## Figures and Tables

**Table 1:** Demographic profile of small towns in the Lodz Province.

		Average	Standard Deviation	Coefficient of Variation [%]
The number of residents				
small towns		7139	5380	75.4
	<5000	2883	733	25.4
	5000–9999	6987	967	13.8
	10,000<	15,273	2754	18.0
Share of people in post-working age				
small towns		25.3	2.0	7.9
	<5 000	24.4	2.3	9.4
	5000–9999	24.9	1.7	6.7
	10,000<	25.9	1.3	5.2
Share of people in pre-working age				
small towns		17.1	1.4	8.2
	<5000	17.5	1.7	9.7
	5000–9999	17.2	1.1	6.5
	10,000<	16.9	1.1	6.6
The old-age dependency ratio (60+)				
small towns		52.9	4.9	9.4
	<5000	51	5.5	10.9
	5000–9999	51.8	4.5	8.7
	10,000<	54.4	3.4	6.2
The feminization rate in the group of older persons (60+)				
small towns		151	10	6.7
	<5000	146	12	8.2
	5000–9999	147	8	5.5
	10,000<	155	5	3.1
Natural movement of the population				
small towns		−5.65	3.32	58.9
	<5000	−5.55	4	72.1
	5000–9999	−5	2.92	58.5
	10,000<	−6.48	2.32	36.3

Source: own study based on the data from the National Census and the Central Statistical Office.

**Table 2:** The respondents’ characteristics.

Characteristics *n* = 500	Total Sample (%)	Characteristics *n* = 500	Total Sample (%)
Sex			
Female	61.8	The number of bicycles owned by household of respondents:	
Male	38.2		
Education			
none	0.2	0	26.0
Primary	4.2	1	25.8
Vocational	16.6	2	30.0
Secondary	44.9	3	10.7
Post-secondary	4.4	4 or more	7.5
Higher	29.7	Household size	
Age			
60–64	28.0	1	31.9
65–69	27.4	2	50.7
70–74	22.8	3 or more	17.4
75–79	14.2	The number of people in age less than 6 years old in household:	
80–84	5.0		
85–89	2.2	0	98.2
90 and more	0.4	1 or more	1.8
Residential building type:		Basic activities (in the last 7 days):	
Single-Family	53.32	unemployed	0.2
Multi-family	46.68	vacation/sick leave	2.8
		work outside the home	17.0
Driving license		hybrid work	0.4
Yes	67.6	home work	2.2
No	32.4	pension	4.0
		retirement	73.3
The number of cars owned by househould of respondents:		Net income per person in the household in EURO: (NPB exchange rate as of 31/10/2022, where 1 Euro = 4.6714 PLN)	
0	9.5	<128.44	1.4
1	47.5	128.44–256.88	8.2
2	17.8	256.88–428.14	41.6
3	3.6	428.14–856.27	38.3
4 or more	1.6	856.27 or more	10.5

Source: own study (Available online: https://euc-word-edit.officeapps.live.com/we/UpgradeBrowser.htm?llcc=pl-PL#_ftn1, accessed on 31 October 2022).

## Data Availability

The data used in this research can be requested by contacting the corresponding author.
